# Early-Stage Diabetic Neuropathy Reduces Foot Strength and Intrinsic but Not Extrinsic Foot Muscle Size

**DOI:** 10.1155/2020/9536362

**Published:** 2020-03-12

**Authors:** Adrienne D. Henderson, A. Wayne Johnson, Lindsey G. Rasmussen, Weston P. Peine, Sydney H. Symons, Kade A. Scoresby, Sarah T. Ridge, Dustin A. Bruening

**Affiliations:** Exercise Sciences Department, Brigham Young University, Provo, UT, USA

## Abstract

**Background:**

Tracking progression of diabetic peripheral polyneuropathy (DPN) is usually focused on sensory nerves and subjective testing methods. Recent studies have suggested that distal muscle atrophy may precede sensation loss. Methods to objectively measure distal muscle size and strength are needed to help understand how neuropathy affects muscle function.

**Purpose:**

To evaluate individual intrinsic and extrinsic foot muscle sizes and functional foot strength in participants with DPN.

**Methods:**

Thirty individuals participated in this cross-sectional study (15 DPN and 15 matched controls). Sizes of 10 separate muscles of the lower leg and foot were measured using ultrasound imaging. Functional foot strength was also quantified using custom great toe and lateral toe flexion tests along with a doming test. Muscle size and strength metrics were compared between groups using ANOVAs and paired *t*-tests (*α* = 0.05). Correlations between strength and relevant muscle sizes were also evaluated.

**Results:**

The sizes of all four intrinsic foot muscles were smaller in individuals with DPN (*p* ≤ 0.03), while only one (toe extensor) of the six extrinsic muscles was smaller (*p* ≤ 0.03), while only one (toe extensor) of the six extrinsic muscles was smaller (*p* ≤ 0.03), while only one (toe extensor) of the six extrinsic muscles was smaller (*p* ≤ 0.03), while only one (toe extensor) of the six extrinsic muscles was smaller (*r* ≤ 0.80) with several corresponding intrinsic muscle sizes. The doming strength test did not show any difference between groups and was moderately correlated with one muscle size (*r* ≤ 0.80) with several corresponding intrinsic muscle sizes. The doming strength test did not show any difference between groups and was moderately correlated with one muscle size (

**Conclusion:**

Diabetic peripheral polyneuropathy affects intrinsic muscles before extrinsics. Ultrasound imaging of individual muscles and functional toe flexion tests can be used clinically to monitor DPN progression and foot function. Participants need to be trained in the doming test before a relationship can be established between this test and DPN foot function. Future studies should include muscle quality measurements to better understand characteristics of affected muscles.

## 1. Introduction

Diabetic peripheral polyneuropathy (DPN) typically affects all three nerve types: autonomic, sensory, and motor; however, clinical diagnosis and assessment have traditionally focused primarily on sensory deficits [1–3]. Many of these sensory tests contain subjective components [4], making it difficult to reliably monitor progression [5]. A research focus on motor function has become increasingly more prominent in scientific literature (e.g., Sacco and Sartor [6]), as signs of atrophy have been shown to precede noticeable sensation loss [7–10]. This atrophy, in part, appears to be due directly to the degenerative effects of DPN as opposed to simple disuse from a more sedentary lifestyle [9, 11]. However, many limitations also exist in muscle evaluations, confining measurements primarily to research settings. For instance, it is difficult to design strength tests that isolate the small muscles of the foot [8, 12], while imaging of muscle size often relies on expensive equipment (e.g., MRI) and burdensome processing time [11]. Simple, inexpensive, and reliable objective measurements for evaluating distal muscle weakness could prove useful in tracking and evaluating motor nerve degeneration as a whole and within specific muscles, further providing insight into DPN progression.

A number of imaging studies have shown general distal muscle atrophy in DPN participants. Typically, when imaging muscles in DPN (with both MRI and ultrasound (US)), only the overall size of lower leg muscle groups or compartments is measured as opposed to individual muscles. Many imaging studies have examined ankle dorsal and plantar flexors [9, 11] and intrinsic [13] or extrinsic [10, 14, 15] foot muscle compartments. This has been done using cross-sectional area (CSA) [8, 10, 13, 16], volume [9, 11], thickness [8, 15], or transverse diameter [8]. All of these studies showed significant decreases in overall lower leg and foot muscle size in DPN as compared to healthy controls. A few studies segmented muscles further by measuring the CSA of the extensor digitorum brevis (EDB) and the thickness or CSA of the first interstitium (area between the first and second metatarsal bones) [7, 8, 17], but that was the extent of the segmentation in DPN. Several studies done on healthy participants [18–20] have imaged up to seven muscles in the lower leg, showing the potential for individual muscle segmentation. The standard for this imaging has been MRI in the past, but ultrasound (US) imaging is less expensive, easier, and faster than MRI [8, 21, 22] and has been used for multiple other pathologies [12, 19, 23]. While image quality is typically higher in MRI, US also has the advantage of capturing muscle contractions using cine-loops, which can help distinguish difficult muscle-fascial borders [24]. The analysis of individual lower limb muscles using US in DPN could be proved useful in monitoring atrophy and motor nerve degeneration.

While US imaging is a promising method for evaluating muscle size, advances in foot and ankle muscle strength measurements can also provide information about motor nerve function in DPN. A number of studies on DPN have used functional strength tests (e.g., isokinetic dynamometer) to evaluate the strength of lower leg muscles [5, 11, 25–28]. These studies have found a decrease in knee and ankle strength that is correlated with neuropathy severity [5, 25, 26] and muscle size [11]. One study also found that the decline in strength in DPN participants was progressive, with annual decreases significantly greater than controls and even other diabetic groups [26]. On the contrary, one study found decreases in strength correlated to severity of neuropathy, but without accompanying decreases in muscle size [27], suggesting that this may be due to intramuscular fat accumulation which would decrease the quality of the muscle but not its size [13, 29, 30]. One limitation of these studies is that they were confined to the ankle dorsal and plantar flexors [5, 11, 25–27] and knee extension and flexion muscles [5, 25, 27]. The strength of the smaller foot muscles has not been evaluated. A few previous studies by our group and others have proposed functional strength testing protocols for evaluating distal foot strength [31–35], but these have not yet been applied to DPN. Foot strength testing in combination with US imaging may provide insight into the manner in which DPN affects atrophy and weakness [12].

The purpose of this study was to assess motor nerve dysfunction in participants with DPN by quantifying muscle size and strength in comparison to healthy controls. We used US imaging to measure individual muscle cross-sectional areas (CSAs) and foot muscle testing protocols to measure functional foot strength. We hypothesized generally that muscle CSAs would be smaller in DPN, with larger deficits in the smaller, more distal muscles. We also hypothesized that muscle strength would be similarly reduced in DPN, and the reductions in strength would correlate with reductions in CSAs. We hope that the specific findings will provide insight into the effects of DPN on motor nerve function and that the methods outlined can eventually be translated to clinical practice to monitor DPN progression.

## 2. Methods

### 2.1. Participants

Data from 15 male participants diagnosed with diabetes mellitus and neuropathy by a physician (DPN) and 15 matched healthy male controls (CON) were included in the study (Table 1). DPN participants were screened for and excluded if they had a history of ulcers, amputation, and any neurological condition besides DPN or could not walk unassisted. CON participants were age-, gender-, and height-matched to the DPN participants. The exclusion criteria for CON included a history of diabetes, any type of peripheral neuropathy, or any lower extremity injury in the past 6 months. All participants were volunteers recruited from the local community and signed consent forms approved by the local ethics board. Each participant's height, weight, and age were recorded, after which the presence of DPN was confirmed using the Michigan Neuropathy Screening Instrument (MNSI) [36].

### 2.2. Protocol

US imaging was performed prior to strength testing to mitigate any acute effects the strength testing might have on imaging results. Due to scheduling conflicts, the order was reversed in a few participants; when this occurred, a rest period was included prior to imaging.

Two US images were taken on 10 muscles of the lower leg and foot by a single experienced researcher, with over 10 years of US imaging experience and an established reliability of 0.99 [24]. US images were collected using a GE Logiq s8 with a 6-15 Hz multilinear probe (most images collected at 10-12 Hz). The following muscles were measured: abductor hallucis (AH), flexor digitorum brevis (FDB), quadratus plantae (QP), extensor digitorum longus (EDL), flexor hallucis brevis (FHB), fibularis longus (FL), fibularis brevis (FB), tibialis anterior (TA), tibialis posterior (TP), and flexor digitorum longus (FDL). Measurements consisted of CSAs for all muscles except for the FHB, where the thickness was used due to concerns over identifying the full muscle borders. The operator was initially blinded to group, but due to participant responses to sensory stimuli during the imaging process and the muscle images themselves, the operator could often determine the group allocation.

**Figure 1:**
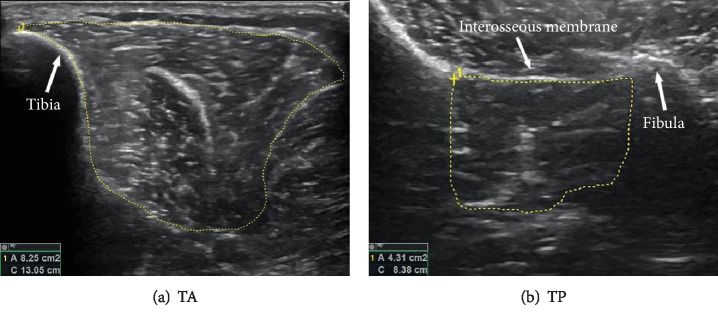
Ultrasound images showing TA (a) and TP (b) measurement borders. The tibia and fibula were used as landmarks with the interosseous membrane in full view to capture the entire circumference of the muscles (yellow outlines).

**Figure 2:**
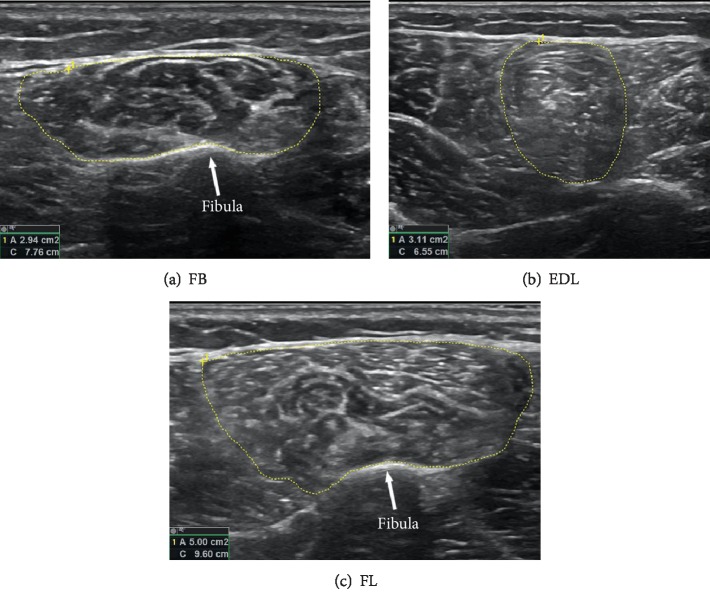
Representative images of the FB (a), EDL (b), and FL (c) measurement. For EDL and FL images, the probe was placed 30% of the way between the lateral joint line and lateral malleolus (laterally for EDL and anteriorly for FL). The probe was placed on the lateral lower leg at a position of 50% between the same landmarks for FB images.

Measurement methodology was based on two previously published papers [37, 38] performed on healthy subjects, and we refer the reader to these papers for additional methodology details, particularly on the intrinsic muscles (FHB, AH, FDB, and QP). Briefly, for these muscles, the participant was seated with the hip flexed and externally rotated, with the knee at 90° and ankle at 30° plantarflexion. This allowed probe access on the medial or plantar foot surface. Each muscle was located using the first metatarsal head (FHB) or navicular tuberosity (AH, FDB, and QP) bony landmarks. For the extrinsic muscles (TA, TP, FDL, EDL, FL, and FB), the hip and knee were slightly flexed, with the foot in a relaxed position (approximately 30° plantarflexion) and a bolster placed under the thigh to eliminate any pressure on the calf. Three of these muscles (FDL, TA, and TP) were also presented in the aforementioned papers. For the FDL, the probe was placed on the medial shank, 50% of the way between the medial knee joint line and medial malleolus. The TA and TP were previously measured as thicknesses; however, with newer equipment and additional experimentation, we were able to measure these as CSAs. The probe was positioned 30% of the way down from the lateral knee joint line to the lateral malleolus. This enabled the fibula and tibia to be visualized with the interosseous membrane in full view, allowing the entire circumference of each of the TA and TP muscles to be seen (Figure 1). We additionally extended our extrinsic muscle measurements to also include the EDL, FL, and FB muscles which were not measured in the previous studies (Figure 2). The EDL and FL were measured with the probe at the same 30% location, positioned on the anterolateral shank for the EDL and rotated slightly laterally for the FL. For the FB, the probe was moved distally to 50% of the way between the same landmarks. When measuring all muscles, the US gain, depth, frequency, and focal point were optimized for visibility. All muscles were first found and isolated while the participant was relaxed, and then the participant contracted the muscle while a cine-loop recorded the movement. This helped us identify the fascial border of each muscle.

**Figure 3:**
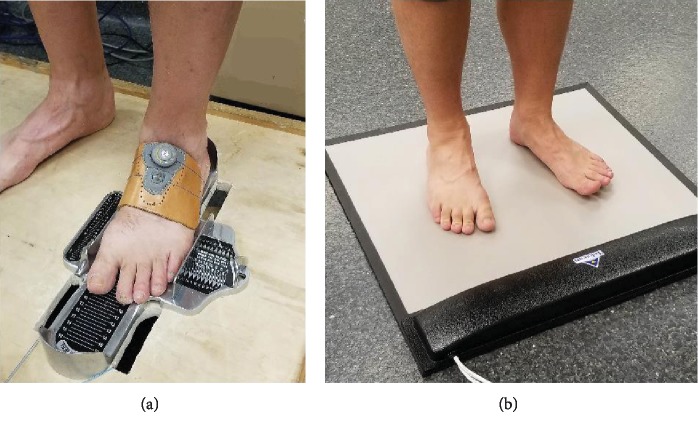
Foot muscle strength testing: (a) DOM test and (b) toe flexion test.

Foot muscle strength testing consisted of three measurements, extracted from two tests. A doming test (DOM) was used to evaluate the muscles controlling the medial longitudinal arch (MLA) while a toe flexion test was used to simultaneously evaluate great toe flexion (GTF) and lateral toe flexion (LTF) strengths. All tests were performed based on previously published methodology [31, 34, 35]. Briefly, the DOM was performed on a custom-built apparatus. Each participant's testing foot was placed in a Brannock device with a leather cuff over the dorsum of the foot. The cuff was attached to a force transducer (Figure 3(a)). Participants performed a short-foot exercise, pulling the ball of the foot towards the heel and pressing upwards on the MLA. Several practice trials were performed prior to testing until the participant felt comfortable with the movement. Three trials of 5 seconds were recorded, with 60 seconds of rest between trials. For GTF and LTF, participants stood on a pressure mat (HR Mat, Tekscan Inc., Boston MA, USA) and pressed downward with all toes, while concurrently extending the contralateral toes in a reciprocal fashion (Figure 3(b)). This test also collected three-five-second duration trials with up to 60 seconds of rest between trials.

### 2.3. Data Analysis

Muscle borders were identified and traced using the US manufacturer-supplied software. Cine-loops and still images were arbitrarily assigned between two trained researchers who drew full muscle boarders and recorded the resulting CSA or thickness calculation (Figures 1 and 2). Study-specific reliability scores (ICC 95% CIs) for these researchers ranged from 0.991 to 0.999. Each border was then retraced, and CSA was recorded by the other trained researcher for consistency. The two images of each muscle were then averaged to get a single measure of muscle size.

Two separate software programs were used to analyze the foot strength data. For DOM, data were recorded and analyzed using a custom LabView (National Instruments, Austin TX, USA) program. Transducer force was plotted over time, and the highest one-second steady-state average force was extracted. Data from the pressure mat was initially processed in Tekscan's Foot Mat software (v. 7.1). Masks were manually drawn around the great toe and lateral toes, and the total force under each of these areas, expressed as a percentage of total force, was exported. Region forces were then imported into the same LabView software and analyzed in the same manner as the transducer forces. All force metrics were averaged across trials.

SPSS v.26 was used for all statistical analyses (IBM Corporation, Armonk, NY). Muscle sizes were compared between groups using one-way ANOVAs with body mass as a covariate. Muscles were also separated into intrinsic and extrinsic groupings, and the total muscle size sum of each grouping was evaluated in the same manner. Muscle strength was expressed in raw force as well as normalized to body mass, and both values were compared between groups using paired *t*-tests. Finally, normalized muscle strength was correlated to muscle size using Pearson's correlation for all subjects (*n* = 30): DOM was correlated with AH, TA, TP, and QP; GTF with FHB; and LTF with QP, FDL, and FDB. Significance was set at *α* = 0.05 for all statistical tests.

## 3. Results

**Figure 4:**
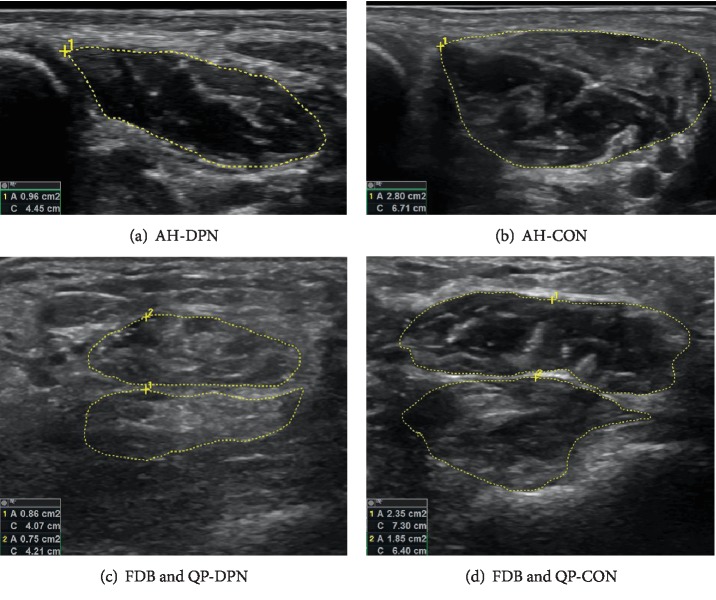
Example comparison of muscle size between DPN (left) and CON (right) subjects. The top row is a comparison of the size of the AH. The bottom row is a comparison of QP and FDB muscles, with FDB being shown above the QP. Values of the circumference (C) and cross-sectional area (A) of the muscles can be seen in the bottom left of each image. (Note that images were only approximately scaled; see numbers in the bottom left corner for area comparisons).

DPN muscle sizes were significantly smaller than CON for five of the ten measured muscles (Table 2). These included all four of the intrinsic muscles (*p* < 0.03) (Figure 4) with an intrinsic muscle sum being 28% smaller for DPN than CON (*p* < 0.001). None of the extrinsic muscles were significantly different between groups except the EDL, which was 6% smaller in DPN (*p* < 0.001). However, the total sum of the extrinsic muscles was significantly smaller in DPN (*p* < 0.037) by approximately the same percentage.

Toe flexor strength tests showed differences between DPN and CON while DOM did not (Table 3). In DPN, LTF was 53.3% smaller in DPN when normalized to BW and 48.9% smaller in raw units, both reaching significance (*p* = 0.005 and 0.006, respectively). GTF was 38% smaller in DPN in raw units, but this was not significant (*p* = 0.058). When normalized to BW, it was 33% smaller in DPN and significantly different (*p* = 0.032).

Only a few muscle size measurements were significantly correlated with strength tests. DOM was not correlated to TA, TP, or AH, but it was moderately correlated to QP (*p* = 0.001, *r* = 0.59). LTF was strongly correlated with QP (*p* < 0.001, *r* = 0.80) and FDB (*p* = 0.001, *r* = 0.60), but not FDL. GTF was only compared to FHB and was found to be moderately correlated (*p* = 0.017, *r* = 0.43).

## 4. Discussion

### 4.1. Muscle Size

The purpose of this study was to evaluate individual foot muscle sizes as well as functional foot strength in patients with DPN. Compared to matched controls, we found a smaller size of all measured foot intrinsic muscles (QP, AH, FDB, and FHB) in DPN (Figure 4); yet, only one of the extrinsic muscles (EDL) was smaller. Our DPN participants were independent ambulators and therefore classified as highly functional and likely in the early stages of neuropathy. Thus, our findings support the clinically observed stocking/glove pattern commonly used to describe DPN progression [39–41], where muscle and nerve degeneration is first observed in the hands and feet followed by the larger muscle groups of the forearm and lower leg, progressing proximally to the upper arm and thigh [42]. It is also possible that the larger muscles of the lower leg compensate for weakness in the smaller intrinsic muscles, helping to preserve the former's size. Additionally, degradation of extrinsic muscle quality (e.g., fatty infiltration) may occur prior to loss of muscle size, explaining functional declines without size changes (see below for further discussion).

While previous studies have shown general lower-extremity muscle atrophy in DPN [9, 11, 43], our findings suggest that early-stage atrophy is primarily confined to the foot intrinsic muscles. This difference in findings could be the result of our individual muscle measurement approach. Measuring the CSA of an entire compartment of extrinsic muscles or multiple compartments as is commonly done [9, 10, 13] can lead to the conclusion that all the muscles of the compartment have atrophied, when the change in CSA is potentially driven by a single muscle. In our case, when the extrinsic muscles' CSAs are summed, they are statistically different between the two groups. However, when looking at each extrinsic muscle individually, this difference appears to be driven primarily by the EDL. A single compartment can contain muscles that perform multiple different actions (e.g., TA inverts the foot while EDL everts the foot). Knowing which specific muscles are atrophying can help therapists target treatment to a particular function and help connect ultrasound images to a compromised functional movement.

### 4.2. Muscle Strength

With some development, the functional strength tests used in this study have the potential to be easy, available tools to assist with DPN diagnosis and progression monitoring in clinical settings. To evaluate the tests used in this study for possible clinical use, we focused on two main aspects of analysis: differences in strength between DPN and CON groups and correlations between these tests and individual muscle sizes. The results from the DOM showed no significant difference between the two groups but a moderate correlation with QP muscle size. This test is meant to isolate the foot intrinsic muscles (AH and FDB) from extrinsic muscles (TA and TP) [44] and is surprisingly not correlated with these other intrinsic muscle sizes. It is possible that the unfamiliarity of this movement combined with the observed intrinsic muscle atrophy in DPN could have resulted in varying intrinsic muscle activation patterns and/or compensations from extrinsic muscles (e.g., TA). Subjects from both groups may need multiple days of practice with this movement to be fully accustomed to it [31]. Due to the lack of strength difference between groups, only a moderate correlation with QP, and the insufficient time to master the movement, further research on DOM is likely needed before it can be used as a clinical tool to diagnose or monitor DPN progression. On the other hand, the GTF test showed strength differences between groups and a moderate correlation with the FHB, while the LTF test showed a significant difference between groups and was strongly correlated with QP and FDB. Surprisingly, this test was not correlated with FDL. This may be because the FDL's contribution to the movement is limited by its smaller CSA when compared to the summed area of QP and FDB, as well as its additional contribution to proximal joint movements including the metatarsophalangeal joints and the ankle. The LTF results in particular (and to a lesser extent GTF) signify that these tests are able to isolate intrinsic muscles and are sensitive enough to show a difference between individuals who have DPN and their healthy counterparts. This simple and inexpensive functional strength test can easily be used in a clinic. Further research should focus on this test's sensitivity to track DPN progression.

### 4.3. Muscle Quality

Past studies have found ankle weakness in participants with DPN without a significant decrease in ankle muscle size [27]. This weakness could be a result of decreased muscle quality, which is a result of metabolic processes (e.g., advanced glycation end products) and decreased use [45]. It is possible that the progression of muscle degeneration within DPN starts with a decrease in muscle quality followed by atrophy. However, there is limited information about this process. Some studies have shown fatty infiltration and increased fibrosity in the large leg muscles of individuals with DPN [13, 27]. Muscle quality has also been investigated in large or easily accessible muscles in various other clinical populations [46–49]. However, no studies have investigated muscle quality in the small leg or foot muscles. Anecdotally, we were able to see a decrease in DPN muscle quality in many of the images of both intrinsic and extrinsic muscles (Figure 4). However, because we sought to maximize morphology measurement by adjusting the frequency and gain of the images for individuals with DPN, the images were not able to be consistently compared for quantitative muscle quality analysis. We plan to conduct future research exploring DPN muscle quality using ultrasound imaging. However, our experience suggests that it may be difficult to find a balance between optimizing settings for clear muscle identification and maintaining consistent settings across subjects for quality comparisons.

### 4.4. Limitations and Future Research

There were a few limitations in this study related to the ultrasound imaging methods. First, due to the FHL tendon location and double muscle head of the FHB, current methodology to obtain a CSA for FHB is dependent on the ultrasound operator to subjectively locate the thickest part of the muscle [19, 50]. We chose a simpler thickness measurement at a uniform distance from the sesamoid bone of the hallux. The use of internal landmarks has been shown to increase accuracy of CSA measurements in comparison to measuring the thickest part of the muscle [19, 24]; therefore, future studies should consider new methodology incorporating internal landmarks with a CSA measurement to increase accuracy and account for different foot lengths if possible. Second, the decreased quality and atrophied nature of many muscles in the individuals with DPN made determination of muscle borders more challenging than for CON. However, variances were similar between CON and DPN group muscle size measurements. Lastly, we did not include all foot intrinsic muscles in our analysis. Additional muscles, especially those with a fibular innervation (e.g., extensor hallucis brevis and extensor digitorum brevis), could help illustrate the progression pattern in DPN [17].

Limitations from the strength testing include the learning curve for DOM as discussed above. Second, correlations between strength tests and muscles sizes were calculated with all participants pooled. Future studies might consider including additional participants to obtain sufficient statistical power to separate the DPN and CON groups for these correlations. Last, it is debatable whether muscle strength measurements should be normalized by body mass. For this reason, we reported strength in both raw units (*N*) and normalized units (%BW), with mostly similar results. Normalization may be preferable as it reduced intersubject variance.

Along with including muscle quality measurements, future research should consider including additional subject groups. Including a wider range of DPN severity will characterize muscle changes as DPN progresses, making it possible for clinicians to monitor their patients' foot muscles more accurately. While we believe our results would be similar for female participants, future research should focus on female recruitment in order to confirm these findings. An additional group of sedentary participants without DPN and a group of participants with diabetes but not neuropathy could ensure that the differences found in muscle size and foot strength are due to the presence of DPN rather than lifestyle or the presence of diabetes.

## 5. Conclusions

By measuring cross-sectional area of foot and lower leg muscles, we were able to see a decreased size in all measured foot intrinsic muscles (QP, AH, FDB, and FHB) but only one extrinsic muscle (EDL), suggesting that DPN primarily affects intrinsic muscles before extrinsic muscles. The GTF and LTF pressure mat strength tests showed differences between groups and were related to specific intrinsic muscle sizes. These tests appear to isolate foot intrinsic muscles from extrinsic ones and show potential as a practical clinical tool for monitoring DPN progression.

## Figures and Tables

**Table 1:** Participant demographics. Comparisons were made between diabetic peripheral neuropathy (DPN) and matched controls (CON) using paired *t*-tests (*α* = 0.05). The presence of neuropathy was confirmed using the Michigan Neuropathy Screening Instrument (MNSI). ∗ indicates a significant difference between groups (*α* = 0.05).

	DPN (*N* = 15)	CON (*N* = 15)	*p* value
Age (yrs)	61.43 ± 12.44	61.64 ± 9.79	0.960
Height (cm)	177.28 ± 7.78	177.99 ± 6.71	0.799
Weight (kg)	103.39 ± 10.08	92.74 ± 15.19	0.103
MNSI score	6.47 ± 2.72	0.27 ± 0.46	<0.001^∗^

**Table 2:** Comparison of muscle sizes between DPN and CON (mean ± SD). All muscles were CSAs with the exception of the FHB. ^∗^Significantly different between groups (*α* = 0.05).

Muscle	DPN	CON	Cohen's *d*	*p* value
Extrinsic muscles				
TA (cm^2^)	7.66 ± 1.25	7.52 ± 1.19	0.11	0.709
TP (cm^2^)	4.28 ± 1.06	4.39 ± 0.97	0.11	0.715
FL (cm^2^)	5.53 ± 1.90	6.46 ± 1.96	0.48	0.142
FB (cm^2^)	4.53 ± 1.18	4.72 ± 1.53	0.14	0.410
EDL (cm^2^)	2.45 ± 0.61	3.16 ± 0.55	1.22	<0.001^∗^
FDL (cm^2^)	1.47 ± 0.52	1.60 ± 0.56	0.24	0.613
*Sum extrinsics*	25.92 ± 2.93	27.86 ± 4.32	0.53	*0.037* ^∗^
Intrinsic muscles				
QP (cm^2^)	1.16 ± 0.30	1.72 ± 0.71	1.03	0.030^∗^
AH (cm^2^)	1.40 ± 0.76	1.96 ± 0.47	0.89	0.023^∗^
FDB (cm^2^)	1.42 ± 0.47	2.16 ± 0.35	1.79	<0.001^∗^
FHB (cm)	1.36 ± 0.18	1.57 ± 0.21	1.07	0.002^∗^
*Sum intrinsics*	5.33 ± 1.25	7.43 ± 1.26	1.67	*<0.001* ^∗^

**Table 3:** Functional foot strength comparisons between DPN and CON (mean ± SD). Doming (DOM), great toe flexion (GTF), and lateral toe flexion (LTF) were expressed in raw units (N) as well as normalized to body mass.

	DPN	CON	*p* value
DOM (%BW)	8.96 ± 5.81	11.72 ± 6.80	0.241
DOM (*N*)	82.03 ± 38.00	99.34 ± 49.66	0.887
GTF (%BW)	5.95 ± 3.95	9.00 ± 3.46	0.032^∗^
GTF (*N*)	57.84 ± 36.22	80.09 ± 28.45	0.058
LTF (%BW)	2.87 ± 1.92	6.14 ± 3.76	0.006^∗^
LTF (*N*)	27.36 ± 16.61	53.55 ± 29.14	0.012^∗^

## Data Availability

The data used to support the findings of this study are available from the corresponding author upon reasonable request.
